# Facile Fabrication of Magnetic, Durable and Superhydrophobic Cotton for Efficient Oil/Water Separation

**DOI:** 10.3390/polym11030442

**Published:** 2019-03-07

**Authors:** Mingguang Yu, Qing Wang, Wenxin Yang, Yonghang Xu, Min Zhang, Qianjun Deng, Guang Liu

**Affiliations:** 1 School of Materials Science and Energy Engineering, Foshan University, Foshan 528000, China; xin15875921595@163.com (W.Y.); yonghangxu@fosu.edu.cn (Y.X.); zhangmin@gic.ac.cn (M.Z.); dqj39@163.com (Q.D.); 2 State Key Laboratory of Pulp & Paper Engineering, South China University of Technology, Guangzhou 510640, China; 3 Sericultural & Agri-Food Research Institute, Guangdong Academy of Agricultural Sciences, Key Laboratory of Functional Foods, Ministry of Agriculture and Rural Affairs, Guangdong Key Laboratory of Agricultural Products Processing, Guangzhou 510610, China; liuguang@gdaas.cn

**Keywords:** cotton fabrics, magnetic, superhydrophobic, oil/water separation, durability

## Abstract

In this paper, we present a facile and efficient strategy for the fabrication of magnetic, durable, and superhydrophobic cotton for oil/water separation. The superhydrophobic cotton functionalized with Fe_3_O_4_ magnetic nanoparticles was prepared via the in situ coprecipitation of Fe^2+^/Fe^3+^ ions under ammonia solution on cotton fabrics using polyvinylpyrrolidone (PVP) as a coupling agent and hydrophobic treatment with tridecafluorooctyl triethoxysilane (FAS) in sequence. The as-prepared cotton demonstrated excellent superhydrophobicity with a water contact angle of 155.6° ± 1.2° and good magnetic responsiveness. Under the control of the external magnetic field, the cotton fabrics could be easily controlled to absorb the oil from water as oil absorbents, showing high oil/water separation efficiency, even in hot water. Moreover, the cotton demonstrated remarkable mechanical durable properties, being strongly friction-resistant against sandpaper and finger wipe, while maintaining its water repellency. This study developed a novel and efficient strategy for the construction of magnetic, durable, and superhydrophobic biomass-based adsorbent for oil/water separation, which can be easily scaled up for practical oil absorption.

## 1. Introduction

Oil spills due to the release of marine oils or industrial wastewater have attracted worldwide attention for their potential pollution to the environment and impact on human health [1,2,3]. Tradition methods such as gravity separation [4], filtration [5], and centrifuge have been used for the separation of organic oils from polluted water. Nevertheless, the above methods are still insufficient in generality, usability and adaptability for oil/water separation owing to the low separation efficiency or complicated operation procedures.

Since Jiang et al. [6] innovatively proposed a straightforward, rapid, and economical method for the construction of steel mesh film with superhydrophobic/superhydrophilic coatings for oil/water separation, various superhydrophobic substrates have gained attention for their potential application in oil/water separation [7,8,9,10,11,12,13,14]. The common oil/water separation materials such as stainless steel [15,16,17], strainer [18,19,20,21], filter paper [22,23,24,25], foam polyurethane [26,27,28,29], fabrics [30,31,32,33,34,35,36] and aerogels [37,38,39] have been widely studied in recent years. There still exist limitations such as low absorption capacities or environmental incompatibility or complicated preparation process.

Cotton is the most abundant and renewable biopolymer, with outstanding properties and various range of applications. The length of cotton fiber is about 20 mm, with a flat structure and a hollow degree of more than 40%. Its special structure advances its use as biomass absorbing material for oily wastewater treatment after hydrophobic modification. Cotton with three-dimensional architecture can achieve a large volume of oil absorption during oil/water separation. However, as far as it is known, there are relatively few reports about cotton fiber directly used as a three-dimensional absorbent material for oil/water separation [40,41,42].

Recently, superhydrophobic materials with magnetic property have attracted wide attention because they can be easily controlled under an exterior magnetic field. Methods have been developed for the fabrication of superhydrophobic magnetic materials by the incorporation of Fe_3_O_4_ magnetic particles due to their inherent low toxicity and strong magnetism [43,44,45,46,47]. However, the reported superhydrophobic magnetic sponges are usually based on polyurethane sponge, polystyrene foam or melamine foam with low absorption capacity and poor recyclability. Besides, the fabricating methods are relatively complicated and time-consuming with multiple steps: preparation of Fe_3_O_4_ nanoparticles, surface treatment of sponge, and dipping or immersing sponge in a solution with Fe_3_O_4_ magnetic nanoparticles and a low-surface-energy compound. Therefore, it is urgent to develop some facile one-step methods with high performance based on biodegradable adsorbents.

This report describes a facile and straightforward strategy to construct superhydrophobic cotton@Fe_3_O_4_ for oil/water separation. The super-wettability surfaces of cotton fabrics were constructed via in situ precipitation Fe^2+^/Fe^3+^ ions onto cotton using PVP as a coupling agent to construct cotton@Fe_3_O_4_ micro/nano hierarchical surface. After surface modification with tridecafluorooctyl triethoxysilane (FAS), the cotton fabrics exhibited not only good superhydrophobic and efficient oil/water separation ability but also fast magnetic responsivity. The prepared cotton also showed mechanical durability against tear and sandpaper friction. Moreover, the adsorption capacity of the cotton could reach more than 50 times its own weight and could be reused for more than 15 cycles.

## 2. Materials and Methods

### 2.1. Materials and Reagents

Cotton was obtained locally. Polyvinylpyrrolidone with average molecular weights of 130 kg/mol (PVP-130), ferric chloride hexahydrate (FeCl_3_·6H_2_O), ferrous chloride tetrahydrate (FeCl_2_·4H_2_O), and tridecafluorooctyl triethoxysilane (FAS, >99%) were obtained from Sigma-Aldrich (Shanghai, China). Ethanol and aqueous ammonia solution (25 wt.%) were supplied by Guangzhou Chemical Reagent Factory (Guangzhou, China). Deionized water was made in-house. The powerful NdFeB magnet (N35, 1.21T) was purchased from Guangzhou Yican Magnetic Material Co., Ltd (Guangzhou, China).

### 2.2. Preparation of PVP-Modified Cotton

Raw cotton was first washed with deionized water and ethanol in sequence to remove dust and then dried at 60 °C for 12 h. Next, 1 g of dry cotton was transferred into 50 mL deionized water containing 0.1 g PVP. After being mechanically stirred at 100 rpm for 30 min, the cotton was then dried at 60 °C for 12 h.

### 2.3. Preparation of Fe_3_O_4_-Modified Cotton

Fe_3_O_4_-modified cotton fabrics were prepared using a modified co-precipitation process [48]. Under gentle magnetic stirring, 1 g PVP-modified cotton was added to 200 mL deionized water containing different amount of ferric chloride and ferrous chloride (varying from 0.54 g/0.19 g, 0.80 g/0.30 g to 1.0 g/0.35 g) at room temperature. After magnetic stirring for 20 min under N_2_ atmosphere, NH_3_·H_2_O solution was added dropwise until the pH reached 8. Then, the mixture was stirred vigorously for 6 h. After being washed in sequence with EtOH and water, the Fe_3_O_4_-modified cotton was dried at 60 °C for 12 h. 

### 2.4. Fabrication of Superhydrophobic Cotton

The solution containing 1.00 mL of tridecafluorooctyl triethoxysilane (FAS) and 99 g of absolute ethanol was mechanically stirred for 2 h. After pre-hydrolysis, the Fe_3_O_4_-modified cotton fabrics were placed in the above solution and mechanically stirred for at least 3 h. The stable water-repellent cotton fabrics were dried at 60 °C for 12 h.

### 2.5. Characterization

Fourier transform infrared spectrometer (FT-IR) was used to analyze structures of cotton before and after surface modification (Spectrum 2000, Perkin Elmer, Waltham, MA, USA). X-Ray photoelectron spectroscopy (XPS) was used to determine the elemental composition of samples (Thermo Electron Escalab 250, Thermo Fisher Scientific Waltham, MA, USA), with Al Ka radiation (20 eV) as the excitation source. A scanning electron microscope (FESEM: Quanta 400F, FEI, Hillsboro, OR, USA) was used to observe the morphological microstructures of samples. The crystallographic phase analysis of samples was performed on an X-ray diffractometer (PW3040/00 X’Pert MPD, Philips, Amsterdam, Netherlands) using Cu-Kα, ranging between 10° and 90°. Thermogravimetric analysis (TGA) was performed on a Q600 instrument with a heating rate of 20 °C/min from 40 to 600 °C under nitrogen atmosphere. A Dataphysics OCA20 contact angle system was used to measure water contact angles (WCAs) of samples with liquid droplets of 5 μL. The oil absorption capacity of samples was determined according to the previously reported method [42].

## 3. Results

**Scheme 1:**
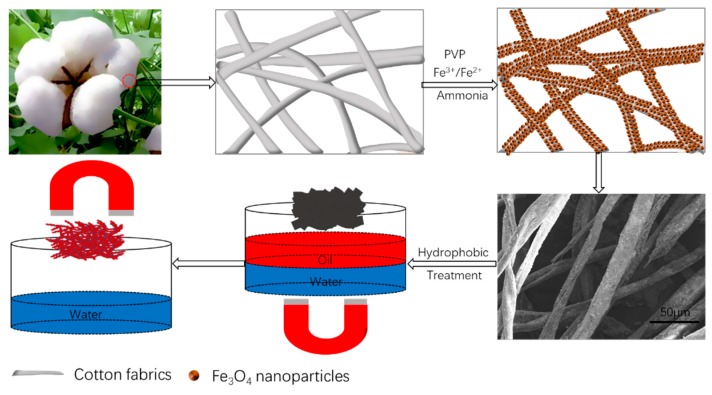
Synthetic procedure for superhydrophobic/superoleophilic Fe_3_O_4_-modified cotton fabrics and subsequent magnetic control of oil/water separation.

Polyvinylpyrrolidone (PVP) is a non-ionic macromolecular compound, which is the most representative among the n-vinyl amide polymers and the most widely studied fine chemicals. PVP can adsorb onto different substrate surfaces, such as polymers, silica, plant fibers, metals and rare earths, attributed to polar -C-N-C=O groups in PVP [49]. This study shows how PVP plays a key role in cotton absorbing Fe^3+^/Fe^2+^ ions and subsequent precipitation in situ forming Fe_3_O_4_-modified cotton. The green procedure for the synthesis of superhydrophobic/superoleophilic Fe_3_O_4_-modified cotton fabrics and following magnetic control of oil/water separation is depicted in Scheme 1. PVP was first grafted onto cotton fabrics, then Fe^3+^/Fe^2+^ ions were adsorbed and subsequently coprecipitated onto cotton surfaces due to the strong static adsorption between Fe^3+^/Fe^2+^ ions and highly polar -C-N-C=O groups in PVP. After hydrophobic treatment with FAS, the cotton fabrics containing Fe_3_O_4_ nanoparticles could be magnetized under external magnetic field, allowing them to be flexibly controlled by a magnet during the oil/water separation process.

**Figure 1:**
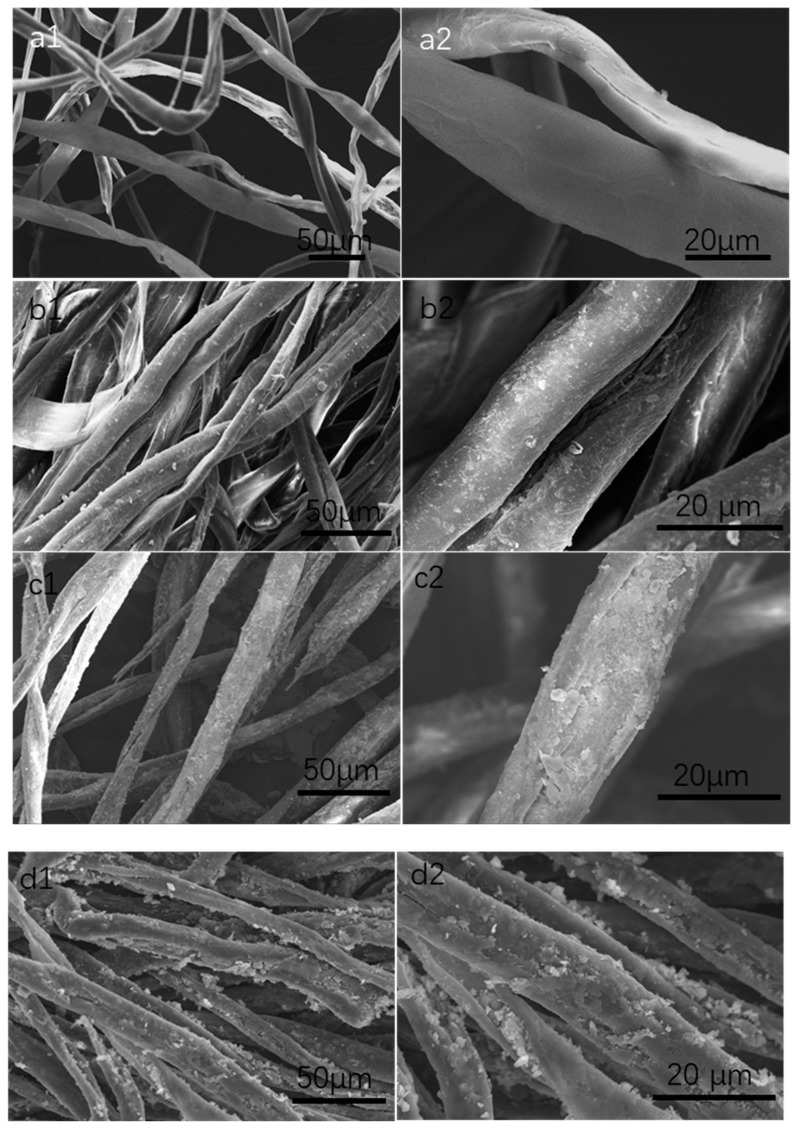
SEM images of raw cotton fabrics (**a1**,**a2**), cotton fabrics modified with different amount of ferric chloride and ferrous chloride (**b1**,**b2**) 0.54 g/0.19 g; (**c1**,**c2**) 0.80 g/0.30 g; (**d1**,**d2**) 1.0 g/0.35 g.

Surfaces of pristine cotton and cotton modified with different amounts of Fe_3_O_4_ were illustrated via SEM analyses. In Figure 1a, raw cotton fabrics had a smooth surface and typical arrangement of fibrils in spiral fashion [50], which were beneficial for loading more Fe_3_O_4_ nanoparticles. After coprecipitation with 0.54 g ferric chloride and 0.19 g ferrous chloride, a thin layer of Fe_3_O_4_ nanoparticles was arranged well on cotton fabrics (Figure 1b). With the amount of ferric chloride and ferrous chloride increased, the amounts of Fe_3_O_4_ nanoparticles on cotton surface increased accordingly. When the addition of ferric chloride and ferrous chloride were 1.0 g and 0.35 g respectively, the Fe_3_O_4_ nanoparticles on the surface of cotton fabrics had a much more heterogeneous coating thickness and a multilayer shell, creating a micro/nanostructured roughness surface (Figure 1d).

**Figure 2:**
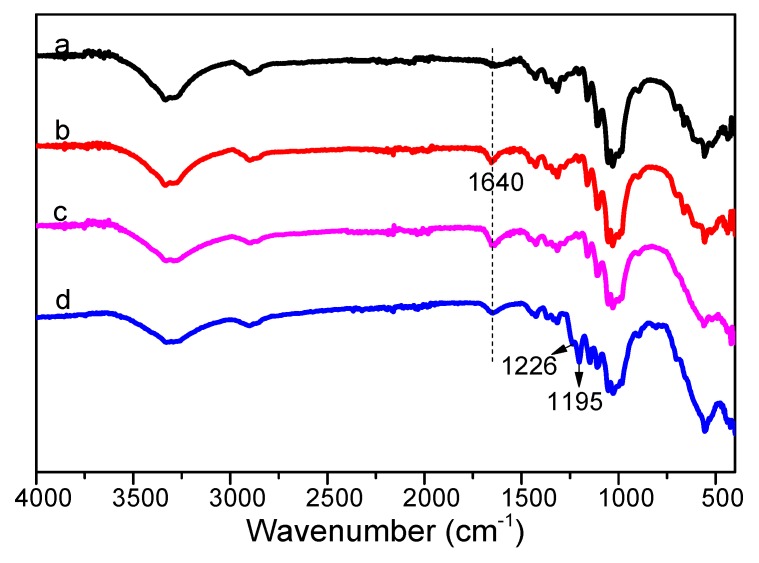
FT-IR spectra of (**a**) raw cotton fabrics, (**b**) PVP-modified cotton fabrics, (**c**) Fe_3_O_4_-modified cotton fabrics, and (**d**) Fe_3_O_4_-modified cotton after fluorination treatment.

FT-IR spectra of the cotton fabrics before and after surface modification in different stages are shown in Figure 2. In Figure 2b, compared with the spectra with raw cotton in Figure 2a, the new vibration peak at 1640 cm^−1^ corresponds to the -C=O group in amide carbonyl of PVP chains, which proved the successful graft of PVP onto the cotton surface. After surface in situ coprecipitation with Fe^2+^/Fe^3+^ ions, the peak around 580 cm^−1^ became stronger due to the stretching vibration of the Fe-O bond in Fe_3_O_4_ in Figure 2c [51]. After hydrophobization treatment of FAS in Figure 2d, new peaks at 1195 cm^−1^ and 1226 cm^−1^ were due to C–F stretching vibrations in FAS [48]. These results indicated that cotton@PVP@Fe_3_O_4_@FAS composites were successfully synthesized to construct magnetic, durable, and superhydrophobic cotton for oil/water separation.

**Figure 3:**
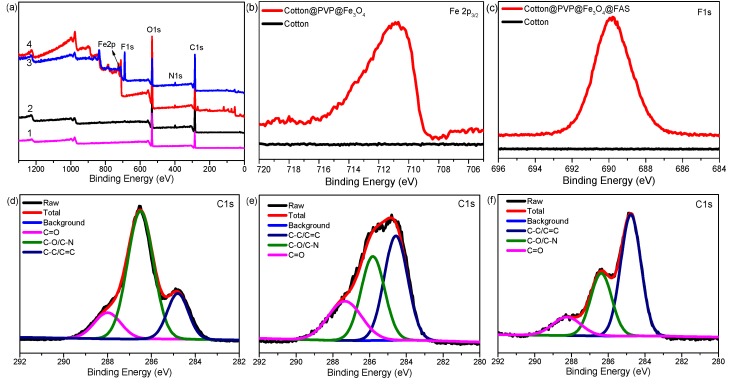
(**a**) XPS survey spectra of pristine cotton fabrics (a1), PVP-modified cotton (a2), Fe_3_O_4_-modified cotton (a3), Fe_3_O_4_-modified cotton after fluorination treatment (a4); (**b**) high-resolution spectra of Fe 2p of pristine cotton and cotton@PVP@Fe_3_O_4_ composites; (**c**) F 1s of pristine cotton and cotton@PVP@Fe_3_O_4_@FAS composites; (**d**) C 1s of pristine cotton fabric; (**e**) C 1s of cotton @ PVP; and (**f**) C 1s of cotton@PVP@Fe_3_O_4_ composites.

XPS spectra were further used to confirm the surface chemical composition of raw cotton fabrics, cotton@PVP@Fe_3_O_4_, and cotton@PVP@Fe_3_O_4_ composites after fluorination (Figure 3). In Figure 3a1, the XPS survey spectra of pristine cotton showed the binding energy of 285.3 eV and 532.6 eV belonging to carbon (C 1s) and oxygen (O 1s) of cotton [52]. In Figure 3a2, a new peak of N 1s in 397.9 eV appeared after modification with PVP. For Fe_3_O_4_-modified cotton and Fe_3_O_4_-modified cotton after fluorination treatment, new peaks at 724.2 eV (Fe 2p_1/2_), 710.3 eV (Fe 2p_3/2_), and 689.1 eV (F 1s) appeared, respectively (Figure 3a3), Figure 3a4, Figure 3b, Figure 3c, which strongly confirmed that nano Fe_3_O_4_ particles were chemically covered on the surface of cotton fabrics and that magnetic superhydrophobic cotton was successfully constructed.

High-resolution spectra of C 1s of pristine cotton, cotton@PVP, and cotton@PVP@Fe_3_O_4_ composites are displayed in Figure 3d–f. In Figure 3d, the C 1s peak was deconvoluted into three peaks of -C=O, C–O, and C–C located at 288.0, 286.5, and 284.8 eV, respectively [53]. After the surface was grafted with PVP (Figure 3e), the relative intensity of C–C/C=C at 284.8 eV and C=O at 288.0 eV increased due to the pyrrole ring in PVP. After surface precipitation of Fe_3_O_4_ (Figure 3f), the intensity of the C=O peak decreased, probably owing to the elimination of the −C–N–C=O of PVP during the deposition of iron ions [54]. These results indicated that PVP acted as a bond bridge for the surface in situ deposition of Fe^3+^/Fe^2+^ and cotton fabrics to synthesize cotton@PVP@Fe_3_O_4_ composites successfully.

**Figure 4:**
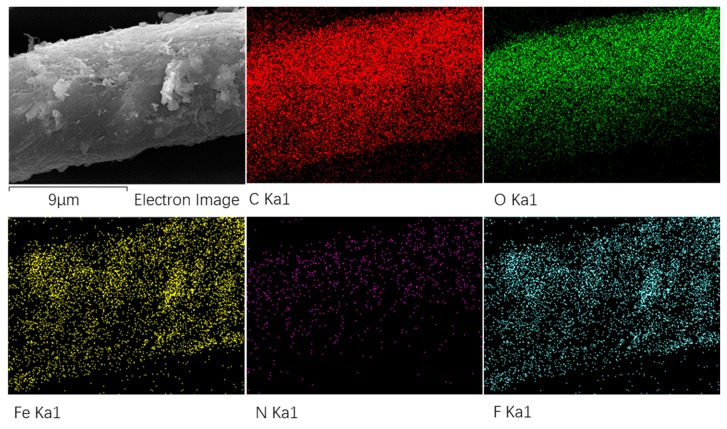
EDS mapping images of Fe_3_O_4_-modified cotton fabrics after fluorination treatment with FAS.

Besides, the surface elemental mapping analysis (EDS mapping) illustrated in Figure 4 demonstrates that each kind of element were uniformly attached to cotton fabrics, which further certified the successful fabrication of cotton@PVP@Fe_3_O_4_@FAS composites.

**Figure 5:**
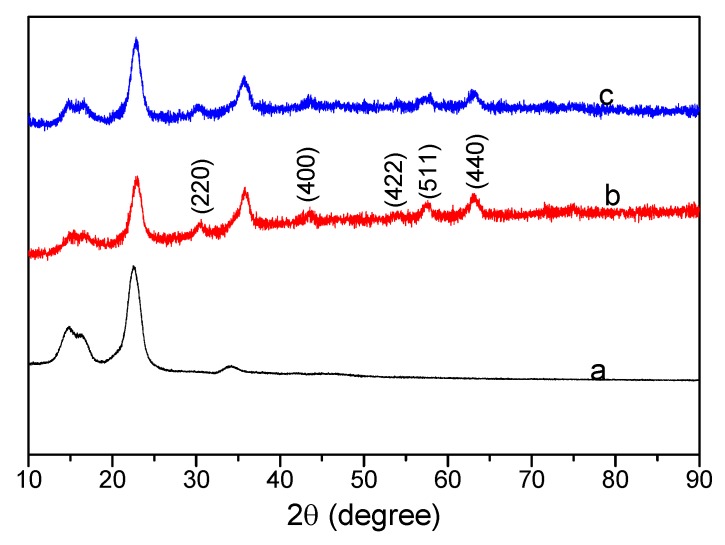
XRD patterns of (**a**) pristine cotton fabrics, (**b**) cotton@PVP@Fe_3_O_4_ composites, and (**c**) FAS-modified cotton@PVP@Fe_3_O_4_ composites.

Figure 5 shows the XRD patterns of pristine cotton fabrics, cotton@PVP@Fe_3_O_4_ composites, and FAS-modified cotton@PVP@Fe_3_O_4_ composites. Figure 5a shows three typical cellulose peaks of raw cotton at 2θ values of 14.6°, 22.4°, and 34.6° corresponding to the reflection planes (101), (002), and (400) [53]. After surface modification with Fe_3_O_4_, there were new diffraction peaks at 2θ of 30.4°, 43.2°, 53.7°, 57.3°, 62.8°, which can be indexed respectively to the (220), (400), (422), (511), and (440) planes of Fe_3_O_4_ [55,56]. The XRD pattern of the FAS-functionalized cotton@PVP@Fe_3_O_4_ composites in Figure 5c was similar to that of cotton@PVP@Fe_3_O_4_ in Figure 5b. The XRD analyses suggested that the surface coprecipitation of Fe_3_O_4_ had little effect on the crystallization property of cotton fabrics. Similarly, hydrophobic modification with FAS of cotton@PVP@Fe_3_O_4_ composites also had little effect on the crystallization property of Fe_3_O_4_ nanoparticles.

**Figure 6:**
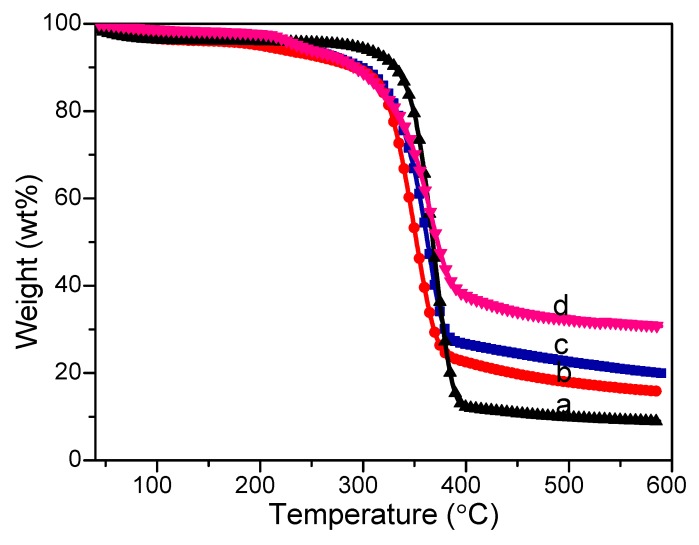
TGA curves of (**a**) pristine cotton fabrics, and Fe_3_O_4_-modified cotton fabrics with different amounts of ferric chloride and ferrous chloride: (**b**) 0.54 g/0.19 g; (**c**) 0.80 g/0.30 g; (**d**) 1.0 g/0.35 g.

The thermal stability and Fe_3_O_4_ amount coated on cotton fabrics were presented in Figure 6. It can be seen that both the raw cotton fabrics and Fe_3_O_4_ modified cotton fabrics with different amount of ferric chloride and ferrous chloride began to decompose at temperatures above 300 °C. This observation showed that Fe_3_O_4_ surface modification did not destroy the chemical structure of raw cotton fabrics. In Figure 6a, the remaining inorganic component of raw cotton fabrics was 8.70 wt% [57]. After surface precipitation of Fe_3_O_4_ particles (Figure 6b), the residual solid slightly increased to 14.79 wt% (Figure 6b. With the addition of ferric chloride and ferrous chloride from 0.80 g/0.30 g to 1.0 g/0.35 g, the residual solid increased from 21.18 wt% to 31.13 wt%, illustrating more Fe_3_O_4_ were attached to cotton fabrics (Figure 6c,d). These TGA curves combined with the SEM analysis in Figure 3 proved that the composites were composed of cotton fabrics and Fe_3_O_4_, which constituted organic–inorganic hybrid composites.

**Figure 7:**
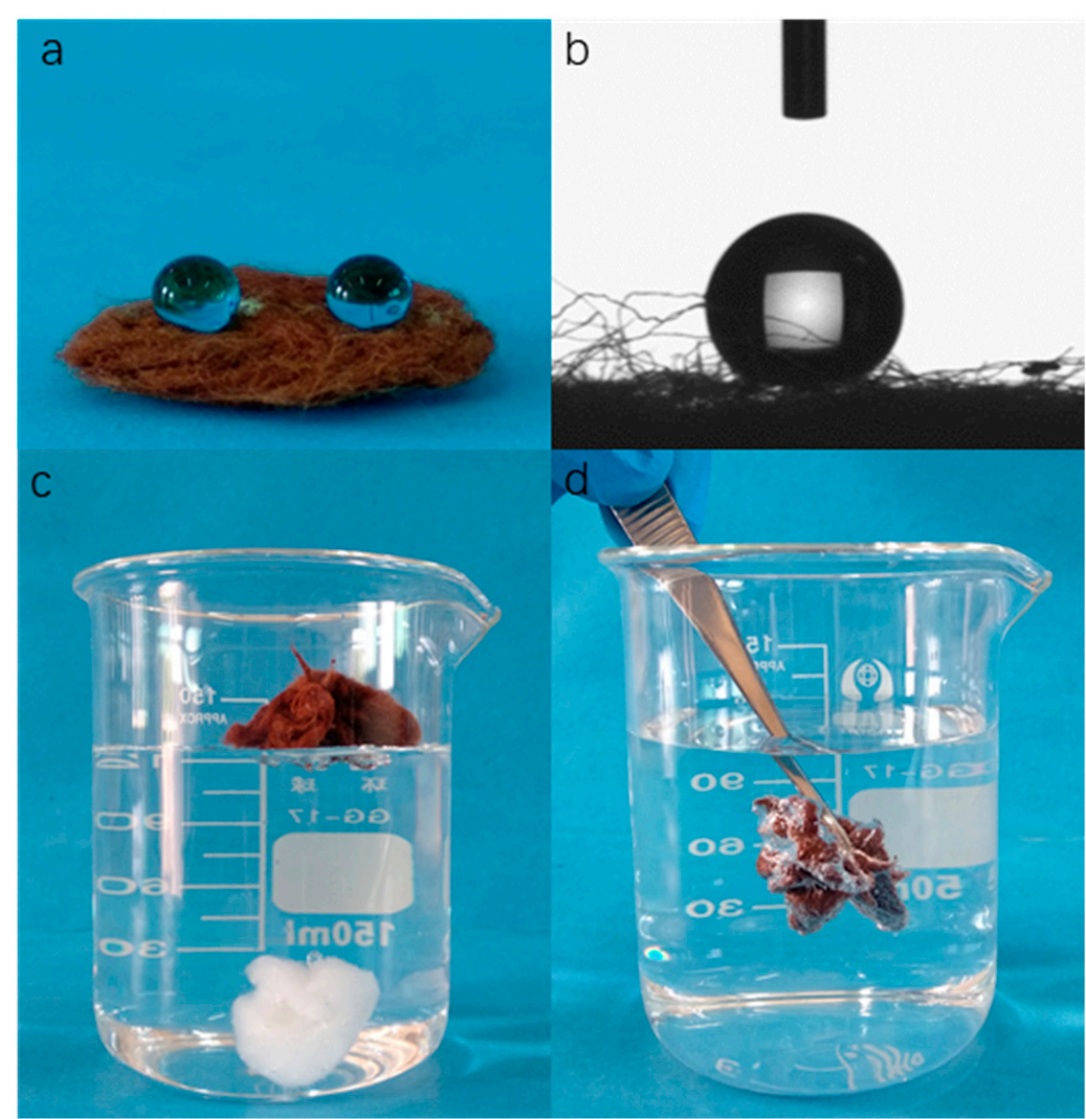
(**a**) Optical images of the water droplets on cotton@PVP@Fe_3_O_4_@FAS surface; (**b**) Shape of a droplet (5 µL) on the as-prepared cotton surface; (**c**) raw cotton and cotton@PVP@Fe_3_O_4_@FAS composites placed in water; (**d**) cotton@PVP@Fe_3_O_4_@FAS composites immersed in water by an external force.

The superhydrophobic properties of the Fe_3_O_4_-modified cotton fabrics were characterized by WCA measurement shown in Figure 7a,b. The as-prepared superhydrophobic cotton fabrics presented excellent superhydrophobic property, with water droplets dyed with methylene blue standing firmly on the cotton fabric’s surface and maintaining spherical shapes with WCA of 155.6° ± 1.2°. After being placed on the water surface, the raw cotton fabrics were quickly wetted, absorbed, and sank into the water, while the modified cotton fabrics still floated on water surface owing to its superhydrophobicity (Figure 7c). Moreover, the silver mirror-like phenomenon could be seen by pressing the modified cotton fiber into the water by an external force (Figure 7d). This nonwetting phenomenon by water was due to the synergistic effect of micro-nano hierarchical structure and low surface energy on the cotton fabrics surface, which was in Cassie–Baxter state [58].

**Figure 8:**
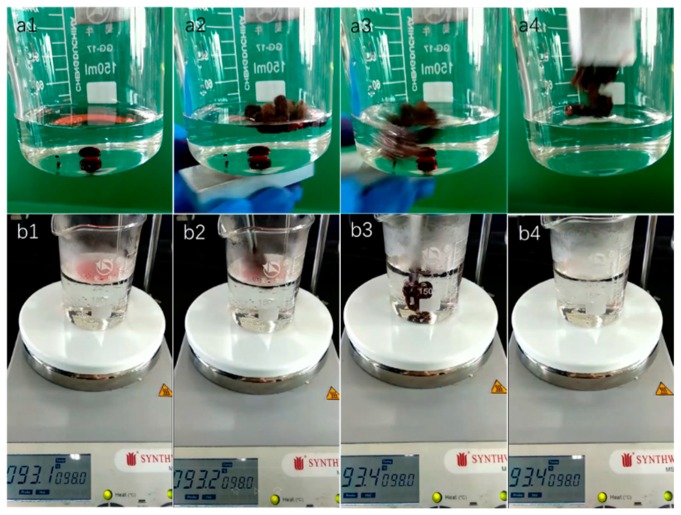
(**a**) Optical images demonstrating the oil/water separation driving by an external magnetic field; (**b**) optical pictures demonstrating the oil/water separation process under hot water at temperatures greater than 90 °C.

The superhydrophobic property of cotton@Fe_3_O_4_@FAS composites endowed them with high-selectivity oil adsorption capacity. Besides, the cotton can be used for oil/water separation under the control of an external magnetic field. In Figure 8a and Video S1, the cotton quickly absorbed the red oil-dyed n-hexane spread on water and the red oil-dyed chloroform from the bottom of the water within several seconds controlled by the external magnetic field. The oil-loaded cotton remained floating on the water’s surface, leaving a transparent region of water and could be taken out of water under magnetic control after oil/water separation, which depicted excellent oil/water separation capability of cotton fabrics. Moreover, the magnetic-responsive cotton also provided a new approach for magnetically controlling the oil/water separation process.

The cotton fabrics also presented durable superhydrophobicity and excellent oil absorption capacity even in hot water at temperatures higher than 90 °C. In Figure 8b and Video S2, the cotton quickly selectively absorbed the n-hexane spread on water, after it was placed into the mixture of n-hexane/chloroform and hot water (>90 °C). When the cotton was immersed into hot water by an external force, it absorbed the red oil-dyed chloroform from the bottom of water within a few seconds. The hot water solution became clean and transparent with the volume nearly unchanged, which showed excellent superhydrophobic stability under high temperature of cotton@Fe_3_O_4_@FAS composites.

**Figure 9:**
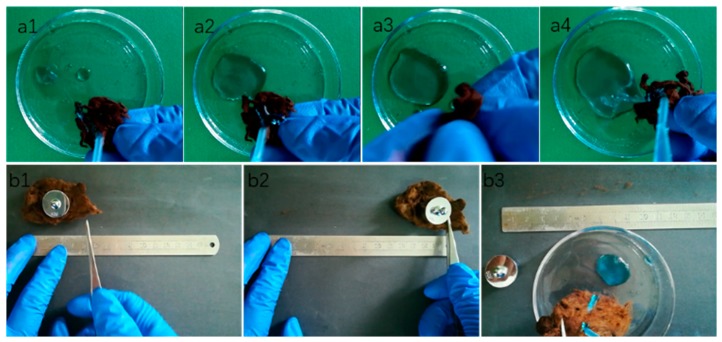
(**a**) Optical pictures demonstrating the method of the finger-wipe test of the as-prepared cotton fabrics; (**b**) optical pictures demonstrating one cycle of the sandpaper-abrasion test of the as-prepared cotton fabrics.

Most reported superhydrophobic materials are inherently fragile to mechanical forces, and even cannot withstand a finger touch owing to their fragile micro/nano hierarchical structure. As a superhydrophobic/superoleophilic oil/water separation material, mechanical stability is of vital importance for the properties and applications of the final oil absorbent material. Therefore, a finger-wipe test was used to evaluate the mechanical durability of the superhydrophobic cotton (Figure 9a and Video S3). After several finger-wipes, rub and tear, the Fe_3_O_4_ nanoparticles remained on the cotton surface with no visible paint loss. Moreover, the water quickly fell off the surface of cotton fabrics, which proved that the cotton still maintained superhydrophobicity.

The friction-resistant experiment against sandpaper in terms of superhydrophobicity, simulating practical conditions, was also tested. As shown in Figure 9b and Video S4, the cotton fabrics were placed on a 400-mesh sandpaper under a weight of 50 g. After several back-and-forth friction cycles, though the sandpaper abrasion could remove the fabrics from the surface of the cotton, and the embedded fabrics were exposed, the cotton still maintained good water repellency. The effect of friction-resistance against sandpaper on the superhydrophobicity of cotton was negligible even after 50 cycles.

**Figure 10:**
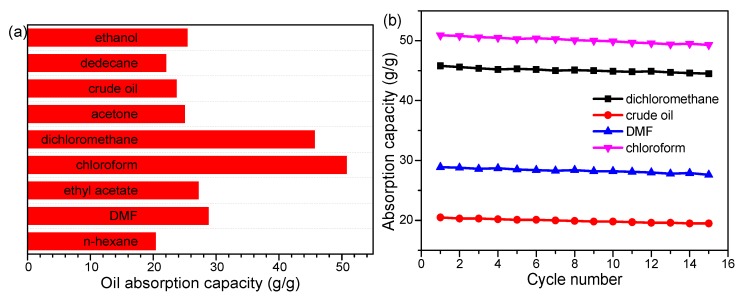
(**a**) Absorption capacity of the as-prepared cotton for different oils and (**b**) reusability of the as-prepared cotton in four different oils.

As an oil/water separation absorbent, the oil absorption ability and durability of the prepared cotton are of great importance for practical applications. To evaluate the oil absorption ability, different kinds of oil were used for the absorption experiment, as shown in Figure 10a. The cotton showed good oil absorption capacity varying from 20.7 g/g to 51.2 g/g depending on different oil densities. Besides, as shown in Figure 10b, the as-prepared cotton also showed good reusability with no obvious decline in absorption capacity even after more than 15 cycles of the absorption process using dichloromethane, crude oil, DMF, and chloroform for study. The results further proved that the as-prepared cotton possesses excellent oil absorption ability for a wide range of oils and mechanical durability even after being reused for 15 cycles, which are quite important properties for actual applications.

## 4. Conclusions

In summary, a facile approach for the fabrication of a magnetic, durable, and superhydrophobic cotton@Fe_3_O_4_@FAS oil absorbent was proposed by in-situ precipitation of Fe^2+^/Fe^3+^ ions onto a cotton surface in the presence of PVP and subsequent hydrophobic modification with FAS. The pyrrolidone ring of PVP could adsorb Fe^2+^/Fe^3+^ and subsequently immobilized the Fe_3_O_4_ nanoparticles on cotton fabrics. The binding of Fe_3_O_4_ nanoparticles on the surface of cotton not only increased the surface roughness but also endowed the cotton with magnetic responsivity. The cotton fabrics exhibited excellent superhydrophobic property, good mechanical and hot water stabilities, and the merits of magnetic actuation. In addition, the cotton fabrics could quickly select absorb oil from water under the control of the external magnetic field, providing new opportunities for the development of novel biomass-based functional adsorbents for oil/water separation.

## Supplementary Materials

The following are available online at https://www.mdpi.com/2073-4360/11/3/442/s1, Video S1: The oil/water separation experiment using the as-prepared cotton fabrics under the control of the external magnetic field. Video S2: The oil/water separation experiment under hot water higher than 90 °C. Video S3: The finger-wipe test to qualitatively evaluate the mechanical durability of the superhydrophobic cotton fabrics. Video S4: The as-prepared cotton fabrics were placed on a 400 mesh sandpaper under a weight of 50 g.
